# Mycobacteriophage Yasnaya_Polyana and its engineered lytic derivative: specificity of regulatory motifs and lytic potential

**DOI:** 10.3389/fmicb.2025.1713073

**Published:** 2025-11-28

**Authors:** Egor Shitikov, Maja Malakhova, Sofya Kuznetsova, Dmitry Bespiatykh, Roman Gorodnichev, Sergei Kiselev, Maria Kornienko, Ksenia Klimina, Aleksandra Strokach, Arina German, Anastasiia Lebedeva, Mikhail Fursov, Anna Vnukova, Dmitry Bagrov, Anastasia Kazyulina, Margarita Shleeva, Marina Zaychikova

**Affiliations:** 1 Lopukhin Federal Research and Clinical Center of Physical-Chemical Medicine of Federal Medical Biological Agency Medicine, Moscow, Russia; 2 Moscow Center for Advanced Studies, Moscow, Russia; 3 Lomonosov Moscow State University, Moscow, Russia; 4 Federal Research Centre ‘Fundamentals of Biotechnology' of the Russian Academy of Sciences, Moscow, Russia; 5 State Research Center for Applied Microbiology and Biotechnology, Obolensk, Russia; 6 Federal State Budgetary Institution “National Medical Research Center of Phtisiopulmonology and Infectious Diseases” of the Ministry of Health of the Russian Federation, Moscow, Russia

**Keywords:** mycobacteriophage, mutagenesis, subcluster K4, start-associated sequences, regulatory motifs, host range, one-step growth curve

## Abstract

**Introduction:**

The growing prevalence of multidrug-resistant *Mycobacterium tuberculosis* and nontuberculous mycobacteria (NTM) highlights the urgent need for alternative therapeutic approaches. Mycobacteriophages, viruses that selectively infect mycobacteria, have emerged as promising tools. Here, we report the isolation and characterization of a new subcluster K4 phage, Yasnaya_Polyana, with a focus on its regulatory motifs and engineered lytic variant.

**Methods:**

The phage was isolated by enrichment on *Mycobacterium smegmatis* mc(2)155, followed by genome sequencing and functional annotation. Start-Associated Sequences (SAS) and Extended SAS (ESAS) were analyzed *in silico* across 188 cluster K phages. A lytic derivative, YPΔ47, was engineered by deleting the repressor gene and characterized in terms of morphology, stability, infection dynamics, and host range.

**Results:**

Yasnaya_Polyana exhibited siphovirus morphology and high genetic similarity with other subcluster K4 phages. Regulatory motif analysis revealed a reduced abundance of SAS and ESAS elements in subcluster K4 phages, including Yasnaya_Polyana, along with specific ESAS sequence deviations. The engineered YPΔ47 mutant retained morphology and infection parameters comparable to the wild-type phage but exhibited a decline in lysogeny frequency (from 18% to <0.01%), confirming a lytic phenotype. Host range analysis revealed limited activity of YPΔ47 against NTM, while the phage demonstrated a high efficiency of plating (EOP = 10^−1^) on *M. tuberculosis* H37Rv and effectively lysed clinical isolates.

**Discussion:**

These findings suggest that Yasnaya Polyana, and apparently other subcluster K4 phages, harbor distinct regulatory features that may reflect divergent transcriptional control strategies. Moreover, YPΔ47 shows potential as a candidate for phage therapy targeting mycobacterial infections.

## 1 Introduction

Bacteriophages (phages)—viruses that infect bacteria—are now re-emerging as promising therapeutic agents against antibiotic-resistant infections. Among these, mycobacteriophages, which specifically target mycobacteria, offer a potential alternative for treating diseases caused by multidrug-resistant *Mycobacterium tuberculosis* and nontuberculous mycobacteria (NTM) (Trigo et al., 2013; Nick et al., 2022; Avdeev et al., 2023; Dedrick et al., 2023).

Mycobacteriophages were first discovered in 1947 (Gardner and Weiser, 1947), but their extensive characterization began in the early 2000s with targeted discovery programs. A major milestone was the launch in 2008 of the Science Education Alliance–Phage Hunters Advancing Genomics and Evolutionary Science (SEA-PHAGES) program (Heller et al., 2024). To date, more than 27,000 phages have been isolated within the program, including over 13,000 that infect mycobacteria.

Comparative genomic analysis of more than 2,000 sequenced mycobacteriophages has grouped them into 31 clusters and several singletons. The vast majority (>99%) were isolated using *Mycobacterium smegmatis* mc(2)155 as a host, a strain that is non-pathogenic, fast-growing, and genetically similar to pathogenic mycobacteria (Malhotra et al., 2017; Sparks et al., 2023). While this model system has been highly productive for phage discovery and characterization, phages capable of efficiently infecting *M. tuberculosis*, the causative agent of tuberculosis, are of particular practical interest. Such phages are mainly represented in clusters G, K, AB, and in subclusters A2 and A3 (Jacobs-Sera et al., 2012; Guerrero-Bustamante et al., 2021), although some singletons also exhibit activity against *M. tuberculosis* (Yang et al., 2024).

Among these, cluster K phages have played a particularly important role in mycobacterial genetics research. Their genetic features, high genomic mosaicism, and ability to infect pathogenic mycobacteria have made them valuable both for fundamental studies and applied purposes. For example, the cluster K phage TM4 has been widely used as a vector for transforming *M. tuberculosis* (Bardarov et al., 1997), in diagnostic systems for tuberculosis detection (Jain et al., 2011), and in assays for determining drug resistance (Piuri et al., 2009).

According to the Actinobacteriophage database (https://phagesdb.org), cluster K currently includes approximately 350 phages, grouped into eight subclusters (K1–K8) based on nucleotide sequence similarity. Genomes of these phages typically contain Start-Associated Sequences (SAS)—short conserved motifs near gene start codons—and Extended SAS (ESAS) elements, which have been proposed to play a role in the regulation of gene expression (Pope et al., 2011; Dedrick et al., 2019a). Cluster K phages can infect both fast- and slow-growing mycobacteria, but infection and lysis efficiency vary greatly among individual phages, and the host ranges of most remain poorly characterized (Jacobs-Sera et al., 2012). Except for TM4, which naturally lost its integration module due to a deletion, cluster K phages are temperate, limiting their direct use in therapy (Pope et al., 2011). However, modern genetic engineering approaches, including Bacteriophage Recombineering of Electroporated DNA (BRED), CRISPR-Cas, and CRISPY-BRED, enable the conversion of lysogenic phages into lytic variants by deleting their integration modules (Pires et al., 2016). For example, the K2 subcluster phage ZoeJ was converted into the lytic derivative ZoeJΔ45 by deleting the repressor gene *gp45* (Dedrick et al., 2019a). Similarly, the subcluster K4 phage Fionnbharth was engineered by removing both the repressor and integrase genes (FionnbharthΔ45Δ47), confirming the feasibility of adapting phages for therapeutic purposes (Guerrero-Bustamante et al., 2021; Dedrick et al., 2023).

In this study, we describe a new mycobacteriophage, Yasnaya_Polyana, belonging to subcluster K4. Based on genome analysis, we performed the first comparative study of SAS and ESAS regulatory sequences across all phages of cluster K and identified distinctive features of subcluster K4, including reduced motif abundance and sequence-specific deviations. We also generated a lytic derivative, YPΔ47, by deleting the repressor gene using the BRED technique. The mutant and wild-type phages were compared in terms of morphological, physicochemical, and biological properties, including their ability to lyse various mycobacterial strains.

## 2 Materials and methods

### 2.1 Bacterial strains and culture conditions

*M. smegmatis* mc(2)155 and *M. tuberculosis* H37Rv, along with clinical isolates of *M. abscessus, M. fortuitum, M. avium*, and *M. kansasii*, were obtained from the collections of the A.N. Bach Institute of Biochemistry (Russian Academy of Sciences) and the National Medical Research Center of Phthisiopulmonology and Infectious Diseases (Ministry of Health of the Russian Federation), respectively.

Clinical isolates of *M. tuberculosis* (369, 463, 1163, 2872, 3431, 8440, and 9211) were provided by the Institute of Tropical Medicine (Antwerp, Belgium) and deposited in the State Collection of Pathogenic Microorganisms (SCPM–Obolensk) under accession numbers B-18239, B-18240, B-18243, B-18244, B-18245, B-18249, and B-18250, respectively. These strains are part of the *M. tuberculosis* drug susceptibility testing panel of the World Health Organization Supranational Reference Laboratory Network.

Mycobacterial strains were cultivated as described previously (Zaychikova et al., 2024) using Middlebrook 7H9 broth (HiMedia, India) supplemented with 0.05% Tween-80 (Sigma-Aldrich, USA) and Middlebrook 7H11 agar (HiMedia, India), both enriched with 10% Middlebrook OADC Supplement (HiMedia, India). Tween-80 was omitted for *M. smegmatis* mc(2)155. For phage isolation and propagation experiments, soft Middlebrook 7H9 agar (0.7%) was used. CaCl_2_ was added to all media to a final concentration of 2 mM for *M. smegmatis* mc(2)155 and 1 mM for other mycobacteria. Cultures were incubated at 37 °C with shaking (for liquid media) in a humidified atmosphere containing 5% CO_2_ unless otherwise specified.

All experiments with pathogenic mycobacteria were performed under Biosafety Level 3 containment in accordance with the biosafety regulations of the Russian Federation. The work was conducted at two authorized facilities: the Culture Collection Department of the State Research Center for Applied Microbiology and Biotechnology (Registration Certificate No. 77ПЧ.01.000.M.000023.04.21, dated 05 April 2021) and the A.N. Bach Institute of Biochemistry, Russian Academy of Sciences (Registration Certificate No. 77.01.16.000.M.000814.03.21, dated 04 March 2021).

### 2.2 Isolation of Yasnaya_Polyana mycobacteriophage

Phage isolation was performed as described previously (Zaychikova et al., 2024) using a poultry yard litter sample collected in the Moscow region. The sample (10 g) was incubated overnight with *M. smegmatis* mc(2)155 (mid-log phase) in 10 mL MP buffer (50 mM Tris–HCl, 150 mM NaCl, 10 mM MgCl_2_, 2 mM CaCl_2_, 0.1 mg/mL ampicillin, pH 7.5). After centrifugation (5,000 g, 10 min) and sequential filtration through 0.45 μm and 0.22 μm PES membrane filters (Millipore, USA), the filtrate was mixed with double-strength TSB broth (HiMedia, India) and inoculated with *M. smegmatis* mc(2)155. The mixture was incubated for 48 h.

Individual phages were isolated using the double-layer agar method (Van Twest and Kropinski, 2009) with three rounds of plaque purification. Phage titers were determined by spot assay as previously described (Sarkis and Hatfull, 1998). Phage lysates were stored at 4 °C.

### 2.3 Construction of a deletion mutant using BRED

The YPΔ47 mutant, carrying a deletion in the CI repressor gene (*yp_00047*), was generated using the Bacteriophage Recombineering of Electroporated DNA (BRED) technique (Marinelli et al., 2019). A 50 bp base sequence (YPΔ47_base) containing two 25 bp flanking regions adjacent to the deletion site was synthesized, along with two pairs of 58 nt extender primers (YPf1/YPr1 and YPf2/YPr2) (Litech, Russia) (Supplementary Table 1). The resulting 200 bp amplicon contained the repressor deletion except for the final 15 codons at the 5′ end. This fragment was purified and concentrated to 50 ng/μL by ethanol precipitation at −20 °C.

A recombinant *M. smegmatis* mc(2)155 strain harboring plasmid pJV53 (HonorGene, China) (van Kessel and Hatfull, 2007) was prepared. Plasmid pJV53 carries the *gp60* and *gp61* genes from mycobacteriophage Che9c, products of these genes enhance recombination frequency. Transformation of electrocompetent *M. smegmatis* mc(2)155 cells with pJV53 was performed under conditions described previously (Parish, 2021) using a MicroPulser (Bio-Rad, USA) at 2.5 kV, 1000 Ω, and 25 μF. The recombinant strain (*M. smegmatis* mc(2)155:pJV53) was stored in 20% glycerol at −80 °C.

For induction of *gp60* and *gp61*, 3 mL of *M. smegmatis* mc(2)155:pJV53 culture were grown for 48 h in the presence of 20 μg/mL kanamycin, then used to inoculate 100 mL medium containing 0.2% succinate and 20 μg/mL kanamycin. Cultures were grown for 18 h to OD_600_ ~ 0.02, after which acetamide was added to a final concentration of 0.2%, and incubation continued for 3 h. Cells were then washed three times with ice-cold 20% glycerol, aliquoted (100 μL), and stored at −80 °C.

Aliquots were co-electroporated with 200 ng of the 200 bp deletion fragment and 50 ng of purified Yasnaya_Polyana phage DNA under the same electroporation conditions. Cells were recovered in 900 μL Middlebrook 7H9 medium supplemented with 2 mM CaCl_2_ for 3 h, then plated in a double-layer agar with 300 μL *M. smegmatis* mc(2)155 (mid-log phase). Plates were incubated for 48 h until primary plaques appeared.

Primary plaques were screened for wild-type or mutant alleles by PCR with primers YPf2 and YPr2 (Supplementary Table 1). Mixed plaques were re-isolated, and secondary plaques were confirmed for the targeted deletion.

### 2.4 Electron microscopy of phage morphology

Phage lysates were filtered through a 0.22 μm PES membrane filter and concentrated by centrifugation for 2 h at 23,000 rpm and 20 °C using an SW 55 Ti swinging-bucket rotor in an Optima XPN-90 ultracentrifuge (Beckman, USA). The resulting pellets were resuspended in 1 mL of SM buffer (50 mM Tris-HCl, 100 mM NaCl, 8 mM MgSO_4_, 0.01% gelatin, pH 7.5) and filtered through a 0.22 μm PES membrane filter. Samples with a titer of at least 10^11^ PFU/mL were used for subsequent analysis.

For TEM imaging, carbon-coated grids (Ted Pella, USA) were pre-treated using a K100X glow discharge device (Quorum Technologies Ltd., UK), and the phage suspension (10 μL) was applied onto the treated carbon surface. After 1-2 min incubation, the suspension was blotted and the grid was stained with a 1% uranyl acetate solution. Visualization was carried out using a JEM-1400 transmission electron microscope (JEOL, Japan) at an accelerating voltage of 120 kV.

### 2.5 Determination of optimal multiplicity of infection

To determine the optimal multiplicity of infection (MOI), *M. smegmatis* mc(2)155 cultures in mid-log phase were diluted in Middlebrook 7H9 broth to a final concentration of 10^7^ CFU/mL and infected at MOIs of 0.001, 0.01, 0.1, 1, and 10. After 48 h incubation, phage titers were determined by spot assay (Sarkis and Hatfull, 1998). All experiments were performed in triplicate.

### 2.6 Phage adsorption assay

The phage adsorption rate to *M. smegmatis* mc(2)155 cells was determined following a previously described protocol (Hendrix and Duda, 1992). Mid-log-phase bacterial cultures were mixed with phage at an MOI of 0.01 in Middlebrook 7H9 medium to a final volume of 5 mL. Aliquots (100 μL) were collected every 10 min over a 60 min incubation, centrifuged at 13,800 g for 2 min, and the supernatant was assayed for unadsorbed phages using the double-layer agar method (Van Twest and Kropinski, 2009). The percentage of unadsorbed phages was calculated and used to generate adsorption curves. All assays were performed in triplicate.

### 2.7 One-step growth curve

The experiment followed a standard protocol (Zaychikova et al., 2024). Briefly, mid-log-phase bacterial cells were infected with phage at an MOI of 0.01 in a total volume of 0.9 mL. After 50 min of incubation, unadsorbed phages were inactivated by adding 0.1 mL ferrous ammonium sulfate (100 mM; Sigma-Aldrich, USA) for 5 min at room temperature. The mixture was centrifuged (13,800 g, 2 min), and the pellet resuspended in 1 mL Middlebrook 7H9 broth.

This suspension was used to determine the number of infected cells using the double-layer agar method (Van Twest and Kropinski, 2009). For growth curve construction, 100 μL of the suspension was diluted 1:100 in fresh 7H9 medium. Aliquots were collected every 30 min for 3 h, centrifuged (13,800g, 2 min), and the free phage titer in the supernatant was determined using the same plating method. Burst size was calculated as the ratio of total released phage particles to the number of initially infected cells. All experiments were performed in triplicate.

### 2.8 Phage stability under different conditions

Phage stability under various pH and temperature conditions was evaluated as previously described (Vandersteegen et al., 2013). For pH stability, 100 μL of phage lysate (5 × 10^9^ PFU/mL for YPΔ47 or 7 × 10^8^ PFU/mL for Yasnaya_Polyana) was added to 900 μL saline adjusted to pH 2–12 and incubated at 25 °C for 24 h. As a control pH 8 at 25 °C was used.

Temperature stability was tested at −20 °C, 4 °C, 20 °C, 37 °C, 45 °C, and 55 °C for 24 h, with 4 °C as the control. In all cases, post-incubation phage titers were determined via spot assay (Sarkis and Hatfull, 1998), and stability was expressed as a percentage relative to the control. All assays were performed in triplicate.

### 2.9 Host range determination

The host range of each phage was assessed by the double-layer agar spot test (Zaychikova et al., 2024). Ten-fold serial dilutions of phage stocks (initial titer: 10^10^ PFU/mL) were prepared in MP buffer. Bacterial test strains were washed twice in Middlebrook 7H9 medium to remove residual Tween-80. Plates were incubated for 48 h for fast-growing mycobacteria and up to 3 weeks for slow-growing strains. Efficiency of plating (EOP) was calculated as the ratio of the phage titer on the test strain to the titer on the propagation host.

### 2.10 Lysogen isolation and superinfection immunity testing

Efficiency of lysogeny assessment, lysogen isolation, and immunity testing were performed following the SEA-PHAGES protocol (https://phagesdb.org/workflow/FurtherDiscovery/).

To evaluate lysogeny efficiency, 100 μL of phage suspension (10^10^ PFU/mL) was evenly spread onto 2% agar plates. Then, ten-fold serial dilutions (10^−3^-10^−7^) of an overnight *M. smegmatis* culture were used as inoculum in top agar, plated onto both phage-coated and control plates, and incubated at 37 °C for 4 days. Lysogeny efficiency was calculated as: Lysogeny % = (CFU on phage plates/CFU on control plates) × 100.

For mc(2)155(Yasnaya_Polyana) lysogeny isolation, serial dilutions of phage lysate were spotted (10 μL) onto double-layer agar plates seeded with *M. smegmatis* mc(2)155 and incubated at 37 °C until bacterial regrowth within lysis zones (mesas) appeared. Bacteria from these zones were streaked onto 2% agar plates, and individual colonies were screened via patch assay for lysogen detection. Positive candidates underwent three rounds of purification and were tested for spontaneous phage release. Integration into the bacterial genome was confirmed by PCR using primers PH-f1, PH-f3, MS-r1, and MS-r2 (Supplementary Table 1) flanking the integration site (Guerrero-Bustamante and Hatfull, 2024), followed by Sanger sequencing of amplicons.

Superinfection immunity was tested against phages Cat (subcluster A3, GenBank acc. No. PQ642456.1), Nadezda (cluster Y, GenBank acc. No. PQ495709.1), Ksenia (cluster S, GenBank acc. No. PQ736089.1), and Vic9 (subcluster B2, GenBank acc. No. PP526940.2). Phage titers and EOP were determined as previously described.

### 2.11 Bacterial growth kinetics Inhibition assay

To assess the impact of mycobacteriophages Yasnaya_Polyana and YPΔ47 on bacterial growth, Middlebrook 7H9 broth, supplemented with OADC enrichment and 2 mM CaCl_2_, was inoculated with 50 μL of a mid-log phase *M. smegmatis* mc(2)155 culture to a final volume of 5 mL. Cultures were incubated at 37 °C with shaking for 18 h until reaching mid-log phase. Subsequently, bacteriophages were added at MOI ranging from 0.01 to 10. Bacterial viability was monitored by determining the number of colony-forming units (CFU) per milliliter at designated time points over a total period of 290 h. Prior to plating, bacterial cells were washed by centrifugation (10 μL aliquot in 1 mL Middlebrook 7H9 broth) to remove free phage particles. All experiments included three independent biological replicates.

### 2.12 DNA extraction and whole-genome sequencing

Genomic DNA of the Yasnaya_Polyana phage and its derivative YPΔ47 was extracted via phenol–chloroform (Green et al., 2012). DNA concentration was measured with the Quant-iT DNA Assay Kit, High Sensitivity (Thermo Fisher Scientific, USA). Libraries were prepared from 100 ng purified DNA using the KAPA HyperPlus Kit (Roche, Switzerland) according to the manufacturer's instructions. Products were purified with KAPA HyperPure Beads (Roche, Switzerland). Library fragment size distribution and quality were assessed using a High Sensitivity DNA Chip (Agilent Technologies). DNA quantification was repeated using the same Quant-iT kit.

Sequencing was performed on Illumina NextSeq 1000 and HiSeq 2500 platforms using HiSeq Rapid PE Cluster Kit v2, HiSeq Rapid SBS Kit v2 (200 cycles), HiSeq Rapid PE FlowCell v2, and NextSeq 1000/2000 P2 Reagents Kit (200 cycles) v3, with 2% PhiX (Illumina, USA) as an internal control.

### 2.13 Bioinformatics analysis

Taxonomic confirmation of the sequenced reads was accomplished with Kraken2 v2.1.2 (Wood et al., 2019) and Bracken v2.8 (Lu et al., 2017). The quality assessment of short paired-end reads was performed using falco v1.2.1 (de Sena Brandine and Smith, 2019) and MultiQC v1.17 (Ewels et al., 2016). Adapters removal and reads filtering was performed using fastp v0.23.4 (Chen et al., 2018). The genome of the Yasnaya_Polyana phage was assembled using Unicycler v0.5.0 and SPAdes v3.15.5. Completeness quality of the assembled phage genome was assessed with CheckV v1.0.1 (Nayfach et al., 2021). Prokka v1.14.6 (Seemann, 2014) and Pharokka v1.7.3 (Bouras et al., 2023) were utilized to annotate the genome's assembly. Subsequently, the annotation was manually curated using GeneMarkS v4.32 (Besemer et al., 2001) and ARAGORN v1.2.41 (Laslett and Canback, 2004). The genome of the phage Yasnaya_Polyana has been deposited in GenBank under accession number PQ495710.1.

To identify polymorphisms in the YPΔ47 mutant, reads were mapped to the wild-type Yasnaya_Polyana genome assembly using BWA MEM v0.7.17-r1188 (Li and Durbin, 2009). Variant calling was conducted using BCFtools v1.1759 (Danecek et al., 2021).

For the phylogenetic analysis, all available cluster K mycobacteriophages (*N* = 188) listed in the Actinobacteriophage database (phagesdb.org; accessed April 30, 2025) were used (Supplementary Table 2). Core gene families for phylogeny were identified with PIRATE v1.0.4 (Bayliss et al., 2019). A maximum likelihood (ML) phylogeny was reconstructed using IQ-TREE 2 v2.3.4 (Minh et al., 2020).

All plots were created in R v4.3.0 (R Core Team. R: A Language and Environment for Statistical Computing. 2023).

The search for SAS and ESAS motifs was performed across the same dataset of 188 Cluster K genomes. For a more detailed characterization, a representative panel of phages from all subclusters was analyzed, including Anaya (JF704106; K1), ZoeJ (KJ510412; K2), Pixie (JF937104; K3), Yasnaya_Polyana (this study), Kratio (KM923971; K5), Unicorn (MF324908; K6), Aminay (MH509442; K7), and Boilgate (MZ274310; K8).

SAS and ESAS motifs were identified using a purpose-built C program (motif_finder.c, available at: github.com/KiselevSI/motif_finder). The search criteria were adopted from Pope et al. (2011). Specifically, SAS motifs were defined as sequences matching the consensus “GGGATAGGAGCCC” with a maximum of two mismatches. ESAS elements were identified as pairs of imperfect 17-nt inverted repeats separated by a variable spacer and located near the SAS motifs. The identified motifs in representative cluster K phages were visualized on genomic maps generated with Phamerator (Cresawn et al., 2011).

*M. tuberculosis* phylogenetic lineages were assigned using the hierarchical scheme described by Shitikov and Bespiatykh (2023) with the tb_mix.py script from the tb-lite pipeline (https://github.com/KiselevSI/tb-lite). Drug resistance was inferred by TB-Profiler (Phelan et al., 2019) from BAM alignments, with all analyses executed within the tb-lite workflow (https://github.com/KiselevSI/tb-lite).

## 3 Results

### 3.1 Genomic characterization of phage Yasnaya_Polyana

The genome of the newly isolated mycobacteriophage Yasnaya_Polyana is a double-stranded DNA molecule 57,978 bp in length, with a GC content of 67% (GenBank accession no. PQ495710). Annotation identified 94 open reading frames (ORFs), collectively spanning 54,336 bp, which corresponds to 93.7% of the total genome length. With the exception of three genes, all ORFs are transcribed in the forward direction.

**Figure 1:**
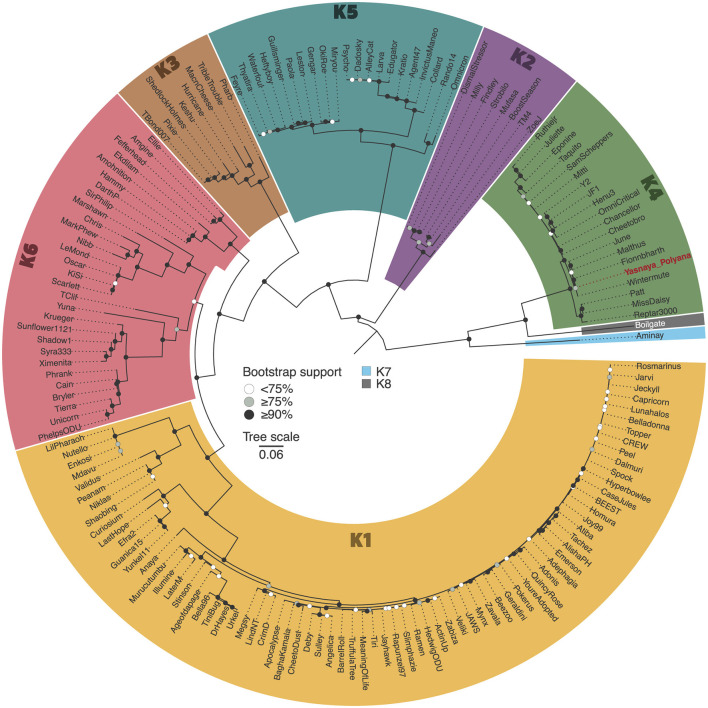
Maximum-likelihood phylogenetic tree of cluster K mycobacteriophages. The midpoint-rooted maximum-likelihood phylogeny was inferred from 162 sequences and 3,121 distinct patterns obtained through core genome alignment of amino acid sequences from cluster K mycobacteriophages. Bootstrap support values are indicated as white dots for values <75%, gray dots for values ≥75%, and black dots for values ≥90% on the interior nodes. Clade colors represent the various subclusters of cluster K. Red colored tip label shows Yasnaya_Polyana phage.

BLASTn analysis revealed that Yasnaya_Polyana shares 94.1% (range: 85.4–99.3%) average nucleotide identity with other phages of the subcluster K4 (genus *Fionnbharthvirus* according to the current ICTV taxonomy [MSL40.v2]). A phylogenetic tree constructed from alignments of core proteins further supports the assignment of the phage to subcluster K4, showing close relatedness to phages Malthus (MN369761.1), Wintermute (MF140435.1), Fionnbharth (NC_027365.1), Cheetobro (NC_028979.1), and June (OR464704.1) (Figure 1).

### 3.2 Functional annotation of the Yasnaya_Polyana genome

**Figure 2:**
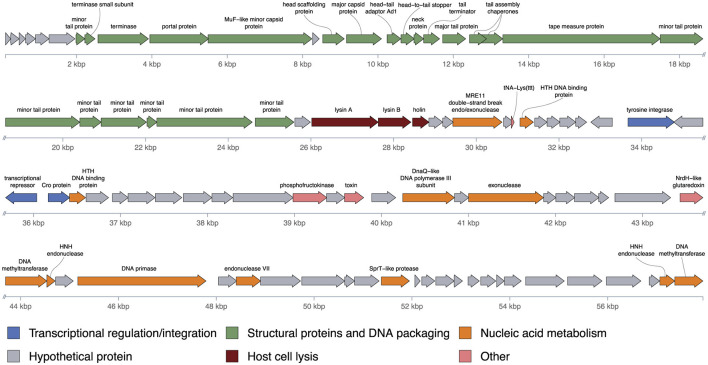
Genomic organization of mycobacteriophage Yasnaya_Polyana. ORFs are represented as colored arrows. Functional annotation is represented by a color code.

Functional annotation classified the encoded proteins into six main categories: (1) structural and virion assembly proteins, (2) host cell lysis proteins, (3) proteins involved in nucleic acid metabolism, (4) proteins associated with integration and lysogeny regulation, (5) hypothetical proteins, (6) other proteins involved in host metabolism and interaction (Figure 2).

Genes encoding structural components for the capsid and tail are clustered in the left portion of the genome, consistent with the genomic organization of siphoviruses (Hatfull, 2014). This region also includes modules for DNA packaging and head-tail joining. Adjacent to these modules are genes encoding the lysis proteins—lysins A and B, and a holin—responsible for host cell degradation. The central part of the genome contains genes involved in lysogeny, including an integrase, a repressor, and a Cro-like protein. The remainder of the genome is dedicated to nucleic acid metabolism, including a DnaQ-like DNA polymerase III subunit, exonuclease, DNA primase, and DNA methyltransferases, alongside a substantial proportion of hypothetical proteins.

### 3.3 Analysis of start-associated and extended start-associated sequences in cluster K phages

The distribution of SAS in the Yasnaya_Polyana genome was characterized by comparative analysis with representative phages from subclusters K1–K8. A total of 125 motifs matching the consensus sequence with ≤ 2 mismatches were identified, of which only six were located within ORFs, indicating a high degree of positional specificity for SAS elements (Supplementary Table 3, Supplementary Figure 1).

Most SAS motifs were located in the right region of the genome, predominantly outside the structural module, consistent with distributions previously described for subclusters K1–K3 (Pope et al., 2011). However, phages from subclusters K4 and K8, which are phylogenetically closely related to each other, showed a pronounced reduction in SAS abundance: Yasnaya_Polyana contained only seven motifs upstream of genes (plus two within coding regions), while the K8 representative contained none (Table S3).

**Figure 3:**
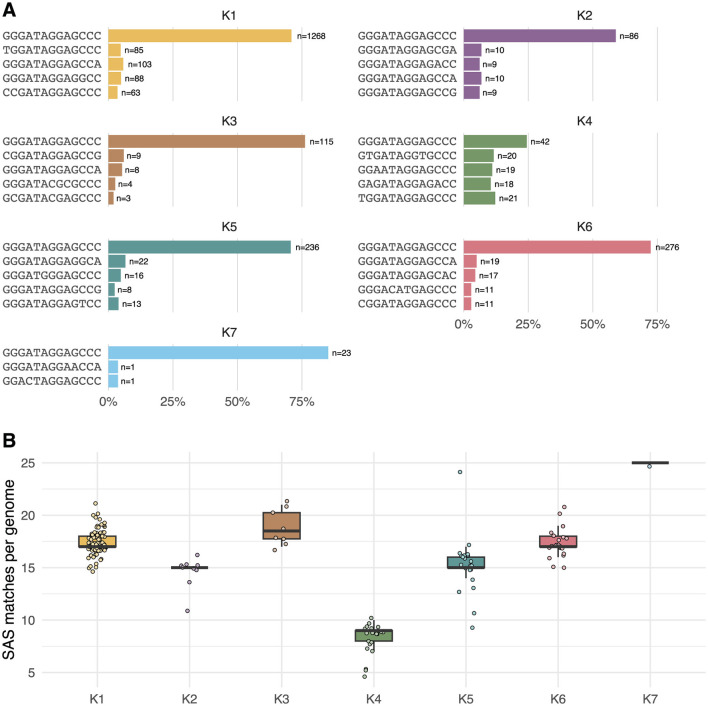
Distribution and frequency of SAS motifs across cluster K mycobacteriophages. **(A)** Number of top 5 scoring SAS motif hits per phage in each subcluster. **(B)** Average number of SAS motifs per genome across subclusters K1–K7. Subclusters K7 and K8 are represented by a single genome each; K8 is not shown due to the absence of detected SAS motifs.

Further analysis across 188 cluster K phages revealed that the frequency of exact matches to the consensus sequence in K1–K3 and K5–K7 phages ranged from 59.7% to 88.4%, whereas subcluster K4 phages exhibited only 22.1% identity (Figure 3A). The mean number of SAS motifs per genome was also lowest in subcluster K4 (9.08) and subcluster K8 (0) (Figure 3B).

To detect potentially divergent SAS forms, sequences with up to three mismatches were examined. Across the eight genomes, 72 such sequences were identified, of which only seven were positioned near start codons rather than within coding regions. Notably, potentially functional motifs were detected in six of the eight subclusters, including one example each in K2, K3, K4, K5, and K7, and two in K8 (Supplementary Table 3, Supplementary Figure 1).

In addition to SAS, a search for ESAS motifs identified 58 such structures across the eight genomes, with none found in the K8 phage (Supplementary Table 3, Supplementary Figure 1). Yasnaya_Polyana again displayed distinct features: of its seven ESAS elements, only one was associated with a SAS motif (one of the two matching the consensus). In three of the seven cases, the last nucleotide of the right repeat coincided with the first nucleotide of the gene start codon. This feature was otherwise observed only in individual representatives of subclusters K5 and K7, where one analogous instance was found in each (Supplementary Table 3).

Another distinctive feature of Yasnaya_Polyana was the presence of thymine at position 14 of the right repeat, a variant not observed in other subclusters (Supplementary Figure 2). The left repeat of Yasnaya_Polyana ESAS motifs resembled that of other clusters; however, position 13 in all Yasnaya_Polyana ESAS contained thymine, a pattern previously described for the K1 phage (Pope et al., 2011) and apparently characteristic of K6 and K7 members. In other subclusters, adenine predominated at this position.

### 3.4 Lytic derivative YPΔ47

The lytic derivative of Yasnaya_Polyana, designated YPΔ47, was generated by targeted deletion of the repressor gene *yp_00047* (positions 35,678–36,037), a conserved target successfully used for generating lytic variants in other cluster K phages like Fionnbharth (K4), Adephagia (K1), and ZoeJ (K2) (Petrova et al., 2015; Dedrick et al., 2019a; Guerrero-Bustamante et al., 2021). The deletion was designed to remove the conserved core domain of the repressor while retaining the initial 45 bp at its 5′ end. This strategy ensured a complete functional knockout, taking into account the ambiguities in the annotation of the precise start codon, which varies among K4 phage genomes.

Whole-genome sequencing revealed, in addition to the deletion, five single-nucleotide polymorphisms (SNPs) in YPΔ47. Three SNPs were located near the recombination site and corresponded to primer-engineered changes introduced to enhance PCR amplification efficiency (Supplementary Figure 3). Another SNP (G33,324T) was detected in the intergenic region between *yp_00044* and *yp_00045*, while the final mutation (C23,660T) resulted in a Thr463Ile substitution in the minor tail protein (*yp_00028*).

**Figure 4:**
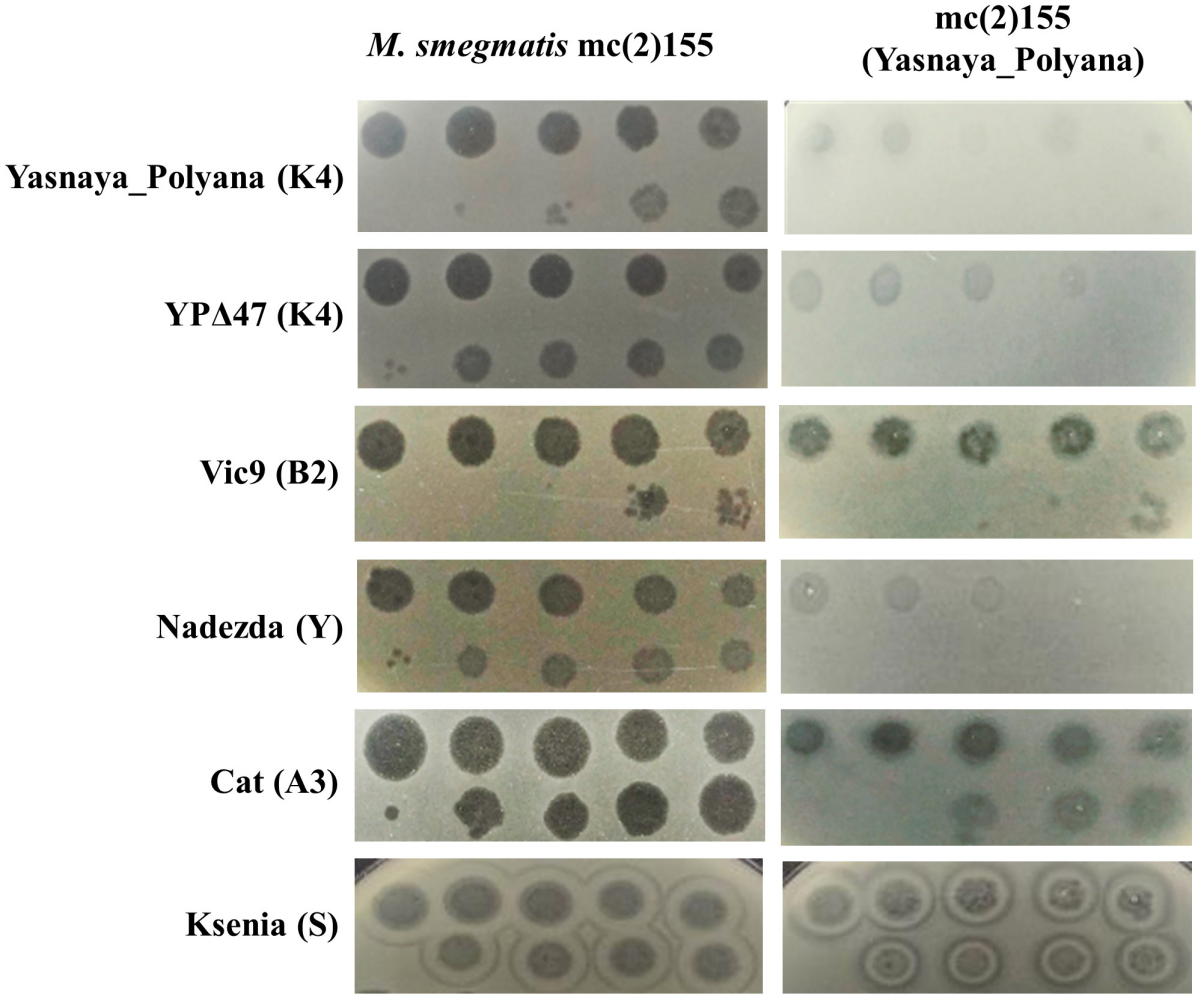
Superinfection immunity profile of *M. smegmatis* mc(2)155(Yasnaya_Polyana) lysogen. Tested phages and their cluster/subcluster affiliations are indicated on the left. Ten-fold serial dilutions of each phage stock (Yasnaya_Polyana: 10^10^ PFU/mL; YPΔ47: 3 × 10^11^ PFU/mL; Vic9: 8 × 10^8^ PFU/mL; Nadezda: 3 × 10^11^ PFU/mL; Cat: 10^11^ PFU/mL; Ksenia: 5 × 10^10^ PFU/mL) were spotted (10 μL) onto lawns of both wild-type *M. smegmatis* mc(2)155 and the *M. smegmatis* mc(2)155(Yasnaya_Polyana) lysogen to assess comparative phage susceptibility.

Lysogeny efficiency assays showed that deletion of *yp_00047* dramatically reduced the ability to establish lysogeny: the lysogen formation frequency for YPΔ47 was <0.01%, compared to ~18% for the wild-type Yasnaya_Polyana. Immunity assays indicated that Yasnaya_Polyana exhibited both homoimmunity and heteroimmunity toward YPΔ47 and a cluster Y phage, but showed no immunity to cluster S, A, or B phages (Figure 4).

### 3.5 Phenotypic characterization

**Figure 5:**
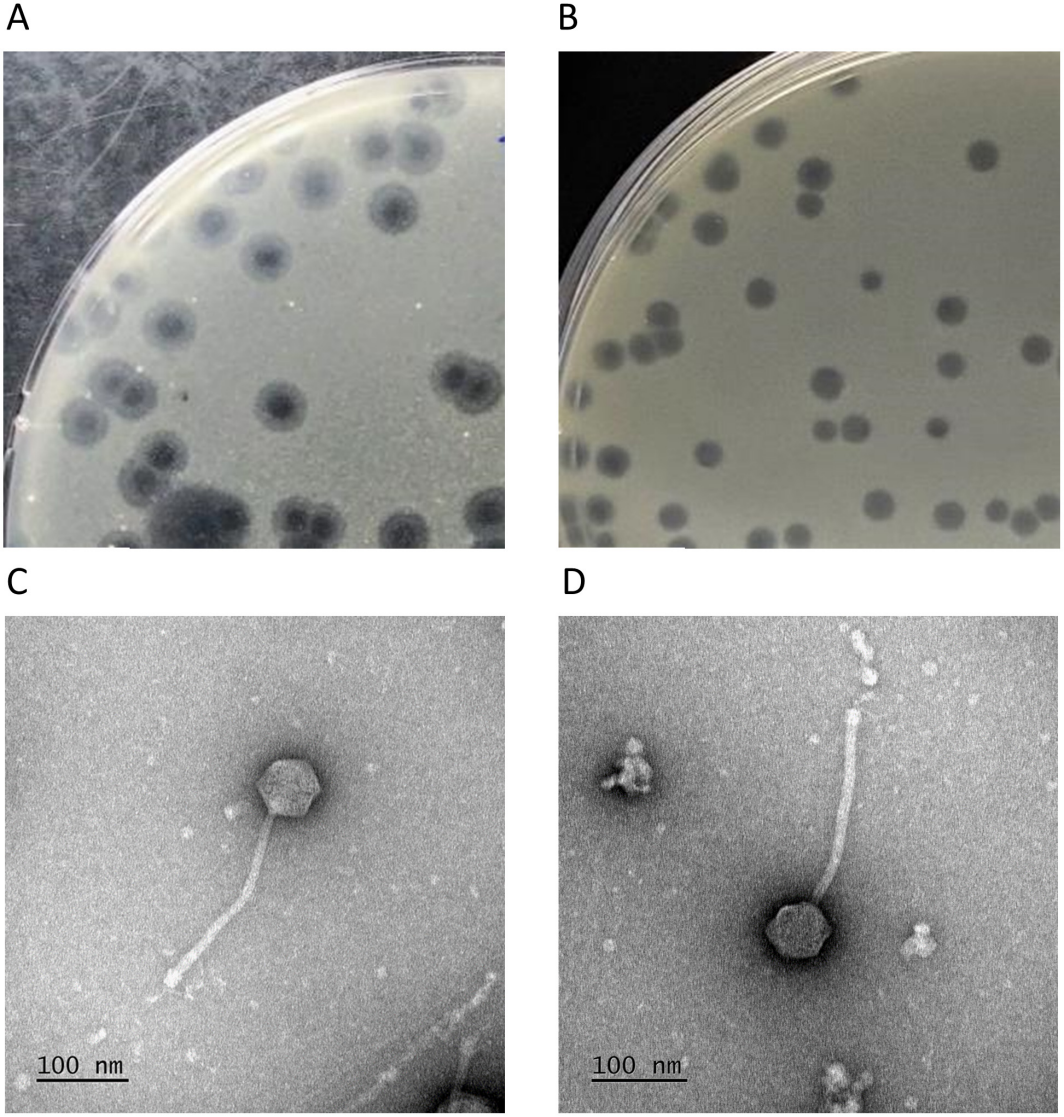
Morphological features of mycobacteriophages Yasnaya_Polyana and YPΔ47. **(A, B)** Plaques formed by Yasnaya_Polyana **(A)** and YPΔ47 **(B)** on a lawn of *M. smegmatis* mc(2)155 after 48 h of incubation. **(C, D)** Transmission electron micrographs of Yasnaya_Polyana **(C)** and YPΔ47 **(D**).

Yasnaya_Polyana and YPΔ47 formed circular plaques 2–4 mm in diameter. Yasnaya_Polyana plaques were characterized by a turbid peripheral ring up to 2 mm wide (Figure 5A), which was absent or weakly expressed in YPΔ47 (Figure 5B). Electron microscopy revealed identical siphoviral morphology for both phages: isometric icosahedral heads (66 ± 3 nm in diameter) and long, noncontractile tails (260 ± 3 nm) with prominent baseplates (Figures 5C, D). In some virions, short lateral tail fibers were visible at the distal tail end.

**Figure 6:**
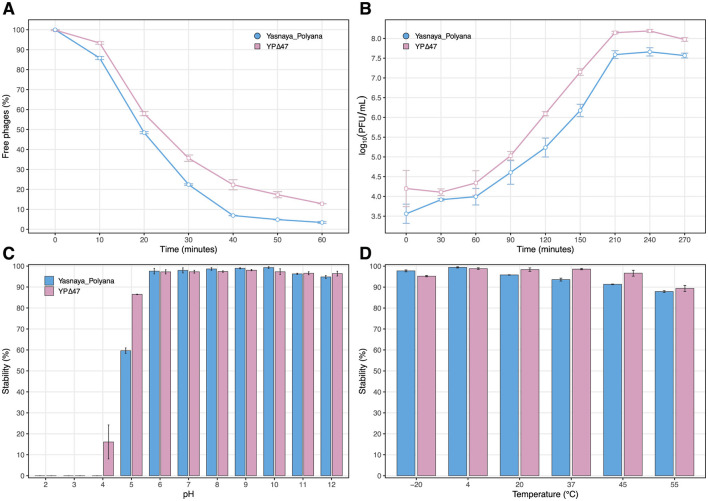
Infection dynamics and stability of mycobacteriophages Yasnaya_Polyana and YPΔ47. **(A)** Adsorption curve. **(B)** One-step growth curve. **(C)** Stability at various pH values. **(D)** Stability at different temperatures.

The optimal MOI for both phages was 0.01, yielding the highest phage titers (~10^9^ PFU/mL). Adsorption curves for Yasnaya_Polyana indicated that most particles bound to *M. smegmatis* mc(2)155 cells within 40 mins of infection (Figure 6A). One-step growth analysis showed a latent period of ~60 mins, followed by a rise period lasting up to 150 mins (Figure 6B). The adsorption and growth curves for YPΔ47 were comparable to those of Yasnaya_Polyana, with burst sizes of 42 and 56, respectively.

Both phages were stable across pH 6–12 and temperatures from −20 °C to 55 °C (Figures 6C, D). YPΔ47 exhibited broader tolerance, with viability decreasing only at pH 4 and temperatures above 45 °C, whereas Yasnaya_Polyana titers declined already at pH 5 and 20 °C.

### 3.6 Bacteriophage-mediated inhibition of bacterial growth

**Figure 7:**
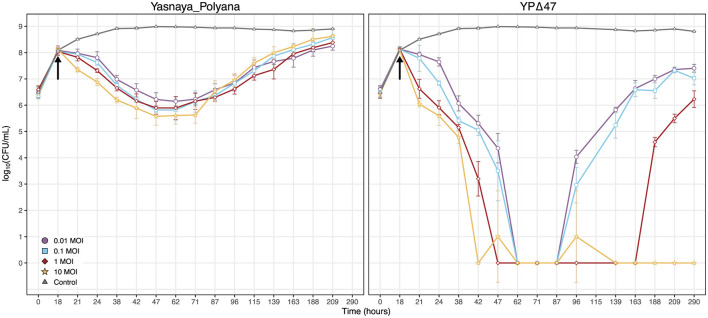
Inhibition of *M. smegmatis* mc(2)155 growth by phages Yasnaya_Polyana and YPΔ47 in liquid culture. Bacterial cultures were infected with the temperate phage Yasnaya_Polyana or its engineered lytic derivative YPΔ47 at the indicated MOI. The black arrow marks the time of phage addition. Data points represent the mean ± SD from three independent biological replicates.

Both Yasnaya_Polyana and its derivative YPΔ47 caused a maximal reduction in bacterial titer of *M. smegmatis* mc(2)155 by 29 h post-infection across the range of MOIs tested (Figure 7). This rapid onset of lytic activity is consistent with the infection dynamics reported for other highly efficient phages, particularly with phage TM4, which also belongs to cluster K (Kalapala et al., 2020). However, the action of YPΔ47 was more pronounced compared to Yasnaya_Polyana, effectively clearing the culture and reducing the bacterial titer to undetectable levels.

Subsequent restoration of bacterial growth in cultures infected with the temperate Yasnaya_Polyana phage, likely due to the formation of lysogens and/or resistant mutants, was initiated after 53 h post-infection, independently of the MOI. In contrast, the dynamics of regrowth for YPΔ47 were dependent on the MOI. At a low MOI, the first signs of bacterial regrowth were observed at 69 h post-infection. This onset of regrowth was delayed with increasing MOI. Notably, at the highest MOI of 10, bacterial regrowth was not detected at any point during the entire 290 h experiment (272 h post-infection), demonstrating a potent and sustained lytic activity.

### 3.7 Host range

**Figure 8:**
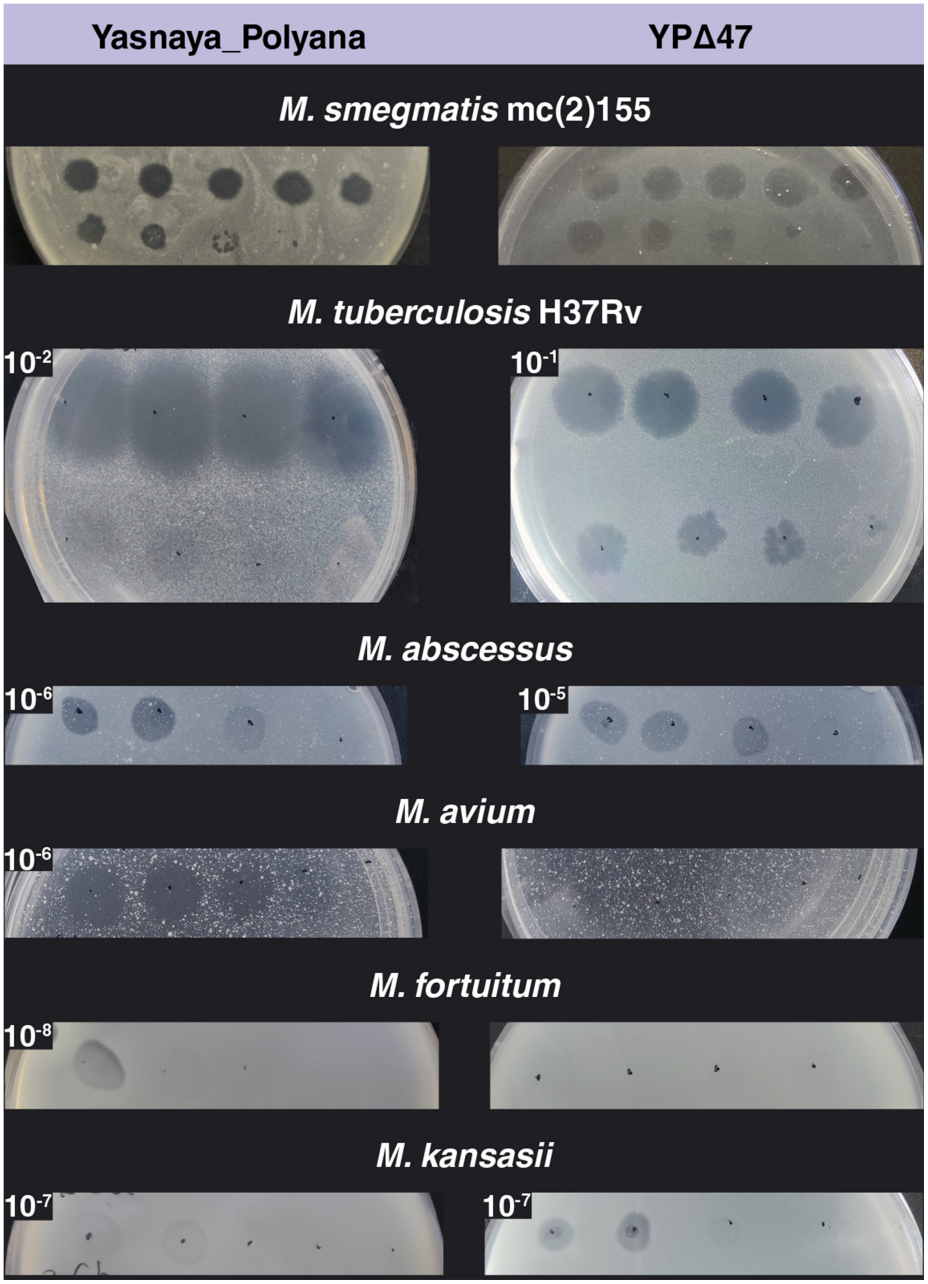
Host range of phages Yasnaya_Polyana and YPΔ47 on different mycobacterial species. Spot assays were performed using serial ten-fold dilutions of the phages (initial titer 10^10^ PFU/mL). EOP values, indicated in the upper left corner of the corresponding image, were calculated as the ratio of the phage titer on the test strain to that on *M. smegmatis* mc(2)155.

Phage Yasnaya_Polyana displayed moderate lytic activity against *M. tuberculosis* H37Rv, producing clear zones of lysis at dilutions up to 10^−3^. The EOP was markedly lower on NTM, with turbid plaques observed for *M. kansasii* and *M. fortuitum* (Figure 8).

Deletion of the repressor in YPΔ47 increased the EOP on *M. tuberculosis* H37Rv by an order of magnitude, producing exclusively clear lysis zones. YPΔ47 lost all activity against *M. avium* and *M. fortuitum*, showed slightly increased activity on *M. abscessus*, and retained unchanged activity against *M. kansasii* (Figure 8). Additionally, YPΔ47 exhibited lytic activity against an extended panel of clinical *M. tuberculosis* isolates belonging to phylogenetic lineages 1, 2, and 4 (Supplementary Figure 4). The phage showed an EOP of 1.0 on two isolates (B-18244 and B-18245) and an EOP of 0.1 on the remaining five, indicating robust infectivity across diverse genetic backgrounds.

## 4 Discussion

In this study, we report the characterization of a new mycobacteriophage, Yasnaya_Polyana, assigned to subcluster K4. The phage exhibits typical siphoviral morphology, and its genomic organization is consistent with other subcluster K4 members. Further comparative analysis with other subclusters confirmed strong conservation of the structural gene module (Supplementary Figure 1). In contrast, non-structural regions were far more divergent, which may underlie the observed differences in regulatory motif distribution. Our *in silico* survey, conducted for the first time across the full set of cluster K genomes (*N* = 188), demonstrated that Yasnaya_Polyana, like other subcluster K4 members, contained substantially fewer SAS motifs than members of subclusters K1–K3 and K5–K7, with one of the lowest proportions of matches to the consensus sequence (Figure 3). Additional distinctive features included a rare co-occurrence of SAS and ESAS motifs and atypical nucleotide positions within ESAS repeats. These findings, in light of the proposed role of SAS and ESAS elements in regulating gene expression (Dedrick et al., 2019a), suggest that subcluster K4 phages may employ a divergent system of transcriptional and translational control.

Despite variation in the distribution of predicted regulatory sequences and other genomic distinctions, Yasnaya_Polyana displayed life cycle parameters comparable to previously described subcluster K1 (Kashi-VT1) and subcluster K4 (Henu3) phages (Nayak et al., 2023; Li et al., 2024). Similarly, it was stable at temperatures up to 55 °C, with reduced viability at lower pH values; its only notable distinction was slightly higher stability under alkaline conditions. The lytic derivative, YPΔ47, exhibited comparable biological characteristics but demonstrated enhanced tolerance to acidic pH and reproducibly greater stability between 20 °C and 45 °C. The mechanisms underlying this increased stability remain unclear, but it is plausible that the absence of the repressor indirectly affects virion physicochemical robustness. Furthermore, growth inhibition assays revealed that while both phages initially suppressed bacterial growth, only YPΔ47 achieved a prolonged bactericidal effect without regrowth. Crucially, this sustained effect was strictly dependent on high MOI, confirming that a stable antibacterial outcome requires not only a strictly lytic phage but also a sufficient initial phage-to-bacterium ratio (Kalapala et al., 2020).

The ~18% lysogeny frequency of Yasnaya_Polyana is consistent with that of other temperate cluster K phages like Adephagia, (K1) and Fionnbharth (K4), and is comparable to the ~20% frequency observed in cluster A phages such as Che12, Bxb1, and L5 (Donnelly-Wu et al., 1993; Sarkis et al., 1995; Jain and Hatfull, 2000; Kumar et al., 2008; Petrova et al., 2015; Guerrero-Bustamante and Hatfull, 2024). By contrast, lysogeny frequencies are generally lower in other clusters: Alexphander (F) exhibits 2.8% (Chong Qui et al., 2024), Giles (Q) forms lysogens at 2–7% (Morris et al., 2008; Dedrick et al., 2013), and similar values of ~5% are reported for clusters I, N, and G (Sampson et al., 2009; Broussard et al., 2013). In comparison, YPΔ47 showed a lysogeny frequency of <0.01%, consistent with other lytic derivatives, such as Adephagia's lytic mutant (0.1%) (Petrova et al., 2015).

The immunity profile of the *M. smegmatis* mc(2)155 (Yasnaya_Polyana) lysogen showed the expected resistance to Yasnaya_Polyana and its derivative YPΔ47, as well as cross-immunity to a cluster Y phage. While the cross-cluster immunity of cluster K lysogens has not been extensively described (Petrova et al., 2015; Dedrick et al., 2019a), similar phenomena have been observed in lysogens from other clusters (Broussard et al., 2013; Chong Qui et al., 2024). These findings suggest that mycobacteriophage immunity mechanisms may extend beyond phylogenetic proximity, underscoring the need for further studies to elucidate the molecular basis and specificity of lysogen defense systems.

Host range assays showed that both Yasnaya_Polyana and YPΔ47 exhibited low activity against the NTM strains tested in this study (*M. abscessus, M. fortuitum, M. avium*, and *M. kansasii*). It is important to note that this assessment was performed on a single representative isolate for each NTM species. As phage sensitivity can vary significantly between clinical isolates within a species (Dedrick et al., 2021), our results do not exclude the potential activity of the studied phages against other NTM isolates of the same species. Nevertheless, the modified variant YPΔ47 showed even lower activity: its ability to infect *M. avium* and *M. fortuitum* was completely lost. This phenomenon may be related to a missense mutation in the *yp_00028* gene encoding a minor tail protein, resulting in a threonine-to-isoleucine substitution at position 463. This change, affecting polarity and side-chain size, could potentially influence receptor recognition or binding efficiency. However, other genetic alterations in YPΔ47, including the deletion of the repressor gene and additional SNPs, may also have contributed to the observed phenotypic shift, either individually or in combination with the tail protein mutation.

On *M. tuberculosis* H37Rv, Yasnaya_Polyana formed turbid lysis zones at dilutions starting from 10^−4^, while YPΔ47 produced clear lysis zones, indicative of a fully lytic infection cycle. The EOP for YPΔ47 was comparable to that of TM4 (K2) and Pixie (K3), though lower than several K1 and K4 phages, including CrimD, Adephagia, Jaws, and Fionnbharth, which have reported EOP values up to 3.0 × 10^1^ (Jacobs-Sera et al., 2012). Importantly, YPΔ47 exhibited strong lytic activity against a panel of clinical *M. tuberculosis* isolates belonging to major phylogenetic lineages (L1, L2, L4), including strains resistant to key modern anti-TB drugs such as bedaquiline, delamanid, and linezolid. This broad activity against diverse and resistant clinical strains is a critical characteristic for a therapeutic candidate and aligns with the profile of other cluster K phages (Guerrero-Bustamante et al., 2021).

Collectively, the potent and broad lytic activity of YPΔ47 against *M. tuberculosis* positions it within a promising cohort of cluster K mycobacteriophages. Notably, engineered lytic derivatives of other cluster K phages, such as ZoeJΔ45 (K2), AdephagiaΔ41Δ43 (K1), and FionnbharthΔ45Δ47 (K4), have shown efficacy against both *M. tuberculosis* and various NTM species, and some have been utilized in compassionate use therapy (Dedrick et al., 2019b, 2021; Guerrero-Bustamante et al., 2021). The inclusion of YPΔ47 in this repertoire provides an additional, genetically distinct tool for constructing tailored phage cocktails. Such cocktails, which combine phages with complementary host ranges—for instance, targeting both *M. tuberculosis* and NTM—are a key strategy in phage therapy to broaden coverage and minimize the emergence of phage resistance (Dedrick et al., 2019b). Therefore, YPΔ47 represents a promising new candidate for developing advanced therapeutic strategies against a spectrum of mycobacterial infections.

While this study provides a comprehensive characterization of the new K4 subcluster phage Yasnaya_Polyana and its engineered lytic derivative, several limitations should be acknowledged. First, although our comparative genomic analysis revealed distinctive features of SAS/ESAS motifs in K4 phages, their functional role in transcription and translation requires further experimental verification. Second, the host range analysis against NTM was assessed on a limited panel of isolates, which may not capture the full spectrum of phage susceptibility present in diverse clinical populations. Third, the lytic derivative YPΔ47 acquired additional genetic modifications, including the Thr463Ile substitution in the minor tail protein that may have contributed to the altered phenotypic properties; the specific contribution of this mutation warrants further investigation.

## 5 Conclusion

This study presents a comprehensive characterization of the new subcluster K4 mycobacteriophage Yasnaya_Polyana, with a particular focus on SAS and ESAS regulatory sequences. Comparative analysis revealed a reduced number of these motifs and distinctive deviations from the consensus in subcluster K4 phages relative to other cluster K subclusters. Such differences may indicate unique mechanisms of gene expression regulation within this subcluster and merit further functional studies in parallel with phages from other groups.

The generation of the lytic derivative YPΔ47 via targeted deletion of the repressor gene reinforces the utility of genetic engineering strategies for converting temperate mycobacteriophages into lytic forms. The mutant retained infectivity, exhibited enhanced stability, and showed high activity against clinical *M. tuberculosis* isolates. These findings highlight the potential of such engineered phages as a foundation for developing phage therapy agents targeting mycobacterial infections, including drug-resistant tuberculosis.

## Data Availability

The data presented in the study are deposited in the GenBank repository (https://www.ncbi.nlm.nih.gov/genbank), accession number PQ495710.1.
