# Supplementary material for: Mycobacteriophage Yasnaya_Polyana and its engineered lytic derivative: specificity of regulatory motifs and lytic potential
Source: Front Microbiol. 2025 Nov 28;16:1713073. doi: 10.3389/fmicb.2025.1713073 (PMC12699233; doi:10.3389/fmicb.2025.1713073)
Supplement: Supplementary file 4 [file Image_1.pdf]

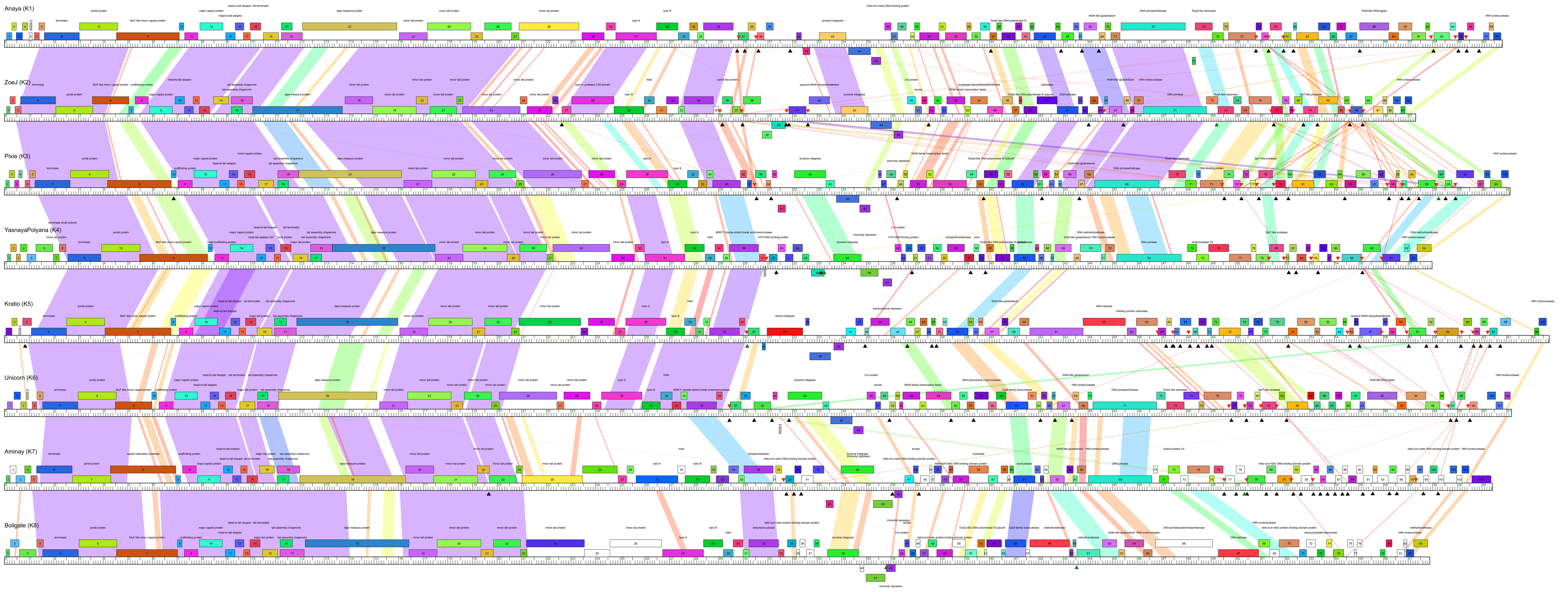

Figure S1. Genome-wide mapping of SAS and ESAS motifs in eight representative cluster K mycobacteriophages. Genomic maps were generated using Phamerator with the “Actino_draft” database (accessed September 2025). Genomes are shown as linear maps with kbp markers. Predicted genes are displayed as colored boxes above or below the genome, corresponding to rightward and leftward transcription, respectively. Gene numbering corresponds to their locus_tag, and putative gene products are indicated above. Triangles denote the genomic positions of motifs: SAS with ≤2 polymorphisms relative to the canonical sequence are shown in black, SAS
