# Supplementary material for: Mycobacteriophage Yasnaya_Polyana and its engineered lytic derivative: specificity of regulatory motifs and lytic potential
Source: Front Microbiol. 2025 Nov 28;16:1713073. doi: 10.3389/fmicb.2025.1713073 (PMC12699233; doi:10.3389/fmicb.2025.1713073)
Supplement: Supplementary file 5 [file Image_2.pdf]

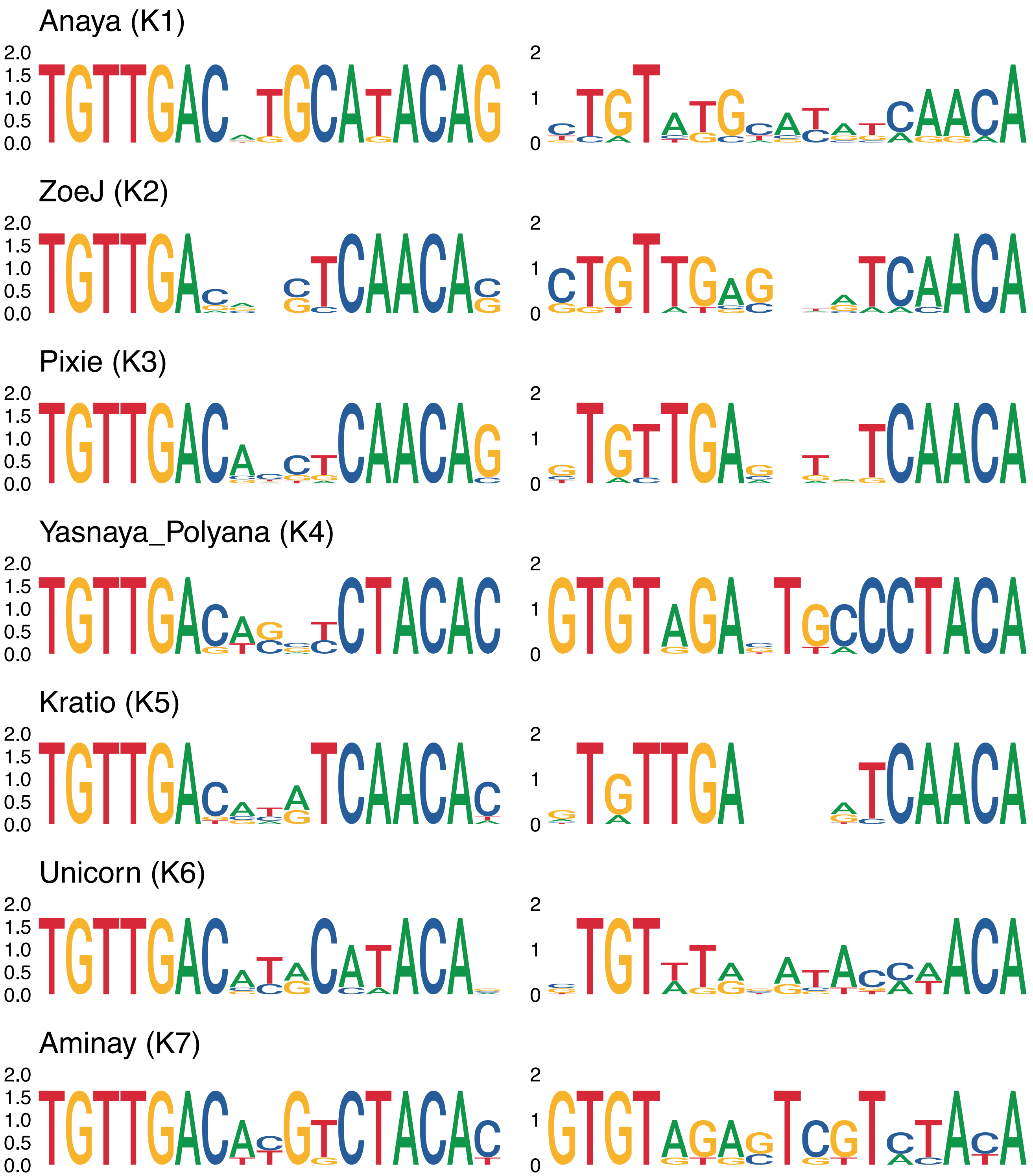


**Figure S2. Sequence logo representation of ESAS motifs across cluster K subclusters.** K8 is not shown due to the absence of detected ESAS motifs.
