# Supplementary material for: Mycobacteriophage Yasnaya_Polyana and its engineered lytic derivative: specificity of regulatory motifs and lytic potential
Source: Front Microbiol. 2025 Nov 28;16:1713073. doi: 10.3389/fmicb.2025.1713073 (PMC12699233; doi:10.3389/fmicb.2025.1713073)
Supplement: Supplementary file 7 [file Image_4.pdf]

M. tuberculosis strain	YPΔ47	EOP	Phylogenetic lineage	Drug resistance profile
**B-18239**	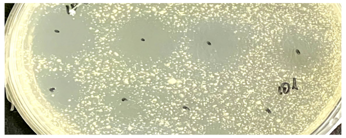	0.1	L4 – L4.3 – L4.3.3	RIF, INH, EMB, PZA, MFX, LVX, AMK, KAN, CAP, ETH
**B-18240**	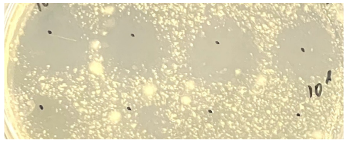	0.1	L2 – L2.2 (modern) – L2.2.M4 – L2.2.M4.7	RIF, INH, DLM, PMD, STR
**B-18243**	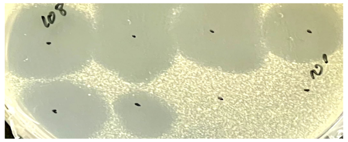	0.1	L2 – L2.2 (ancient)	BDQ
**B-18244**	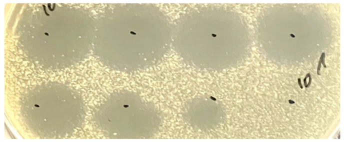	1	L2 – L2.2 (modern) – L2.2.M2 – L2.2.M2.5	LZD
**B-18245**	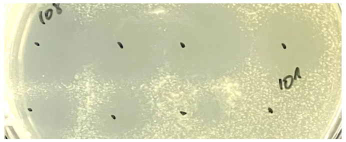	1	L4 – L4.9	LZD
**B-18249**	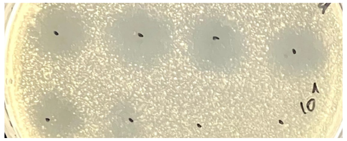	0.1	L1 – L1.1 – L1.1.3	DLM, PMD
**B-18250**	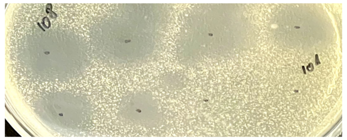	0.1	L2 – L2.2 (modern) – L2.2.M4 – L2.2.M4.9 – L2.2.M4.9.1	RIF, INH, EMB, PZA, STR, AMK, KAN, CAP

Figure S4. Lytic activity of the YPΔ47 phage against a panel of drug-resistant clinical isolates of M. tuberculosis. Serial ten-fold dilutions of the phage lysate (initial titer 108 PFU/mL) were spotted onto lawns of different clinical isolates. The classification of phylogenetic lineages follows the hierarchical scheme described in Shitikov and Bespiatykh (2023). The tested panel includes multidrug-resistant isolates belonging to major M. tuberculosis phylogenetic lineages: L1 (Indo-Oceanic), L2 (East Asian), and L4 (Euro-American). Drug resistance profiles were determined using TB-Profiler (Phelan et al., 2019). Abbreviations for antibiotics: RIF, Rifampicin; INH, Isoniazid; EMB, Ethambutol; PZA, Pyrazinamide; MFX, Moxifloxacin; LVX, Levofloxacin; BDQ, Bedaquiline; DLM, Delamanid; PMD, Pretomanid; LZD, Linezolid; STR, Streptomycin; AMK, Amikacin; KAN, Kanamycin; CAP, Capreomycin; ETH, Ethionamide.
