# Supplementary figures and images for: Mycobacteriophage Yasnaya_Polyana and its engineered lytic derivative: specificity of regulatory motifs and lytic potential
Source: Front Microbiol. 2025 Nov 28;16:1713073. doi: 10.3389/fmicb.2025.1713073 (PMC12699233; doi:10.3389/fmicb.2025.1713073)

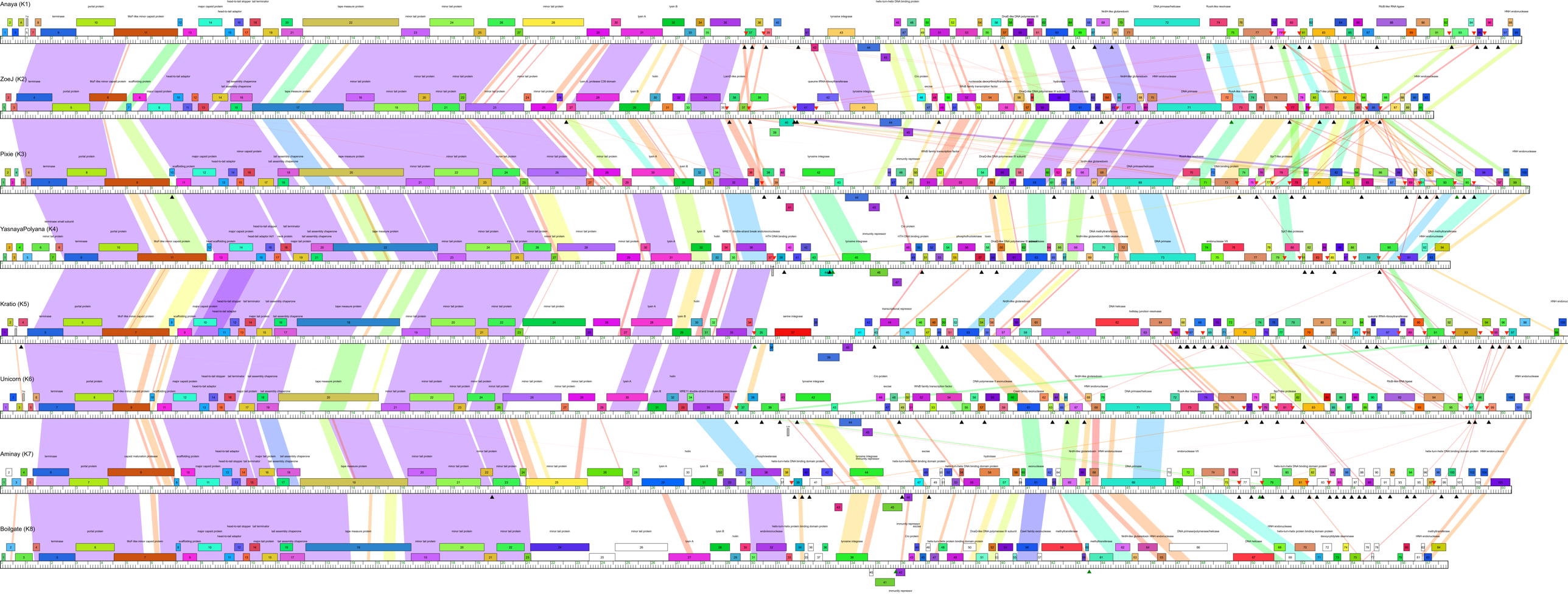

Supplement: Supplementary file 8 [file Data_Sheet_1.pdf]

Anaya (K1)

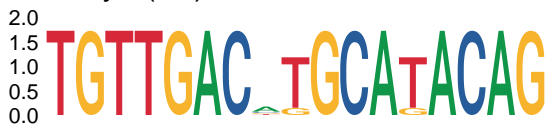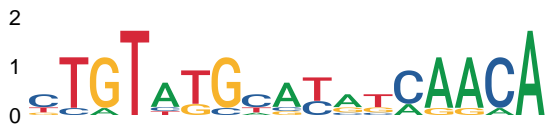

ZoeJ (K2)

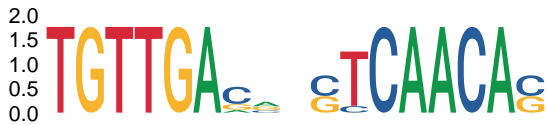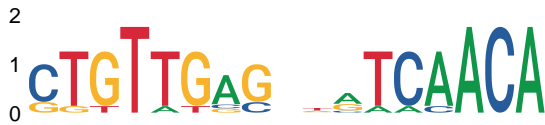

Pixie (K3)

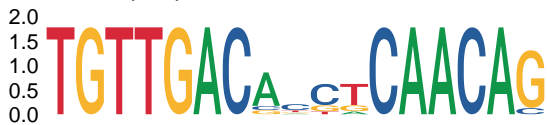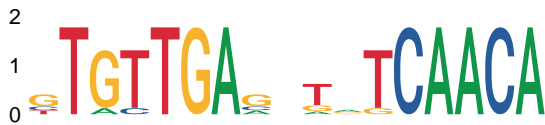

Yasnaya\_Polyana (K4)

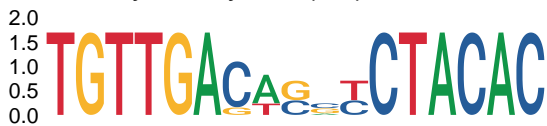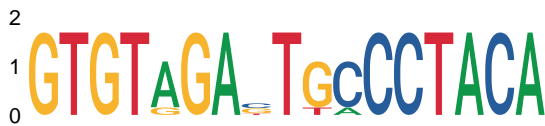

Kratio (K5)

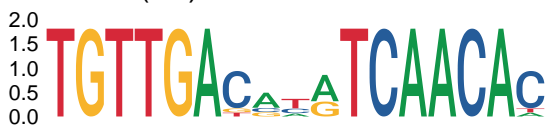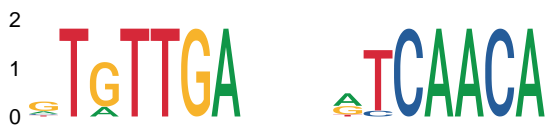

Unicorn (K6)

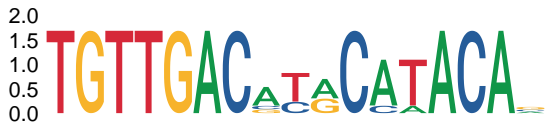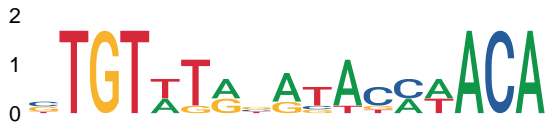

Aminay (K7)

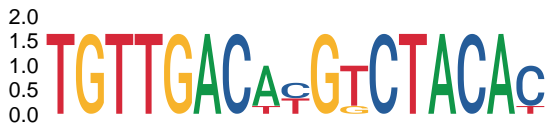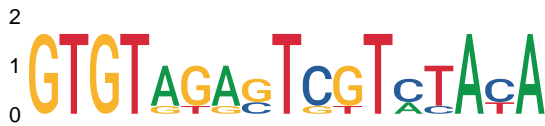

Supplement: Supplementary file 9 [file Data_Sheet_2.pdf]
