# Supplementary material for: Mycobacteriophage Yasnaya_Polyana and its engineered lytic derivative: specificity of regulatory motifs and lytic potential
Source: Front Microbiol. 2025 Nov 28;16:1713073. doi: 10.3389/fmicb.2025.1713073 (PMC12699233; doi:10.3389/fmicb.2025.1713073)
Supplement: Supplementary file 6 [file Image_3.pdf]

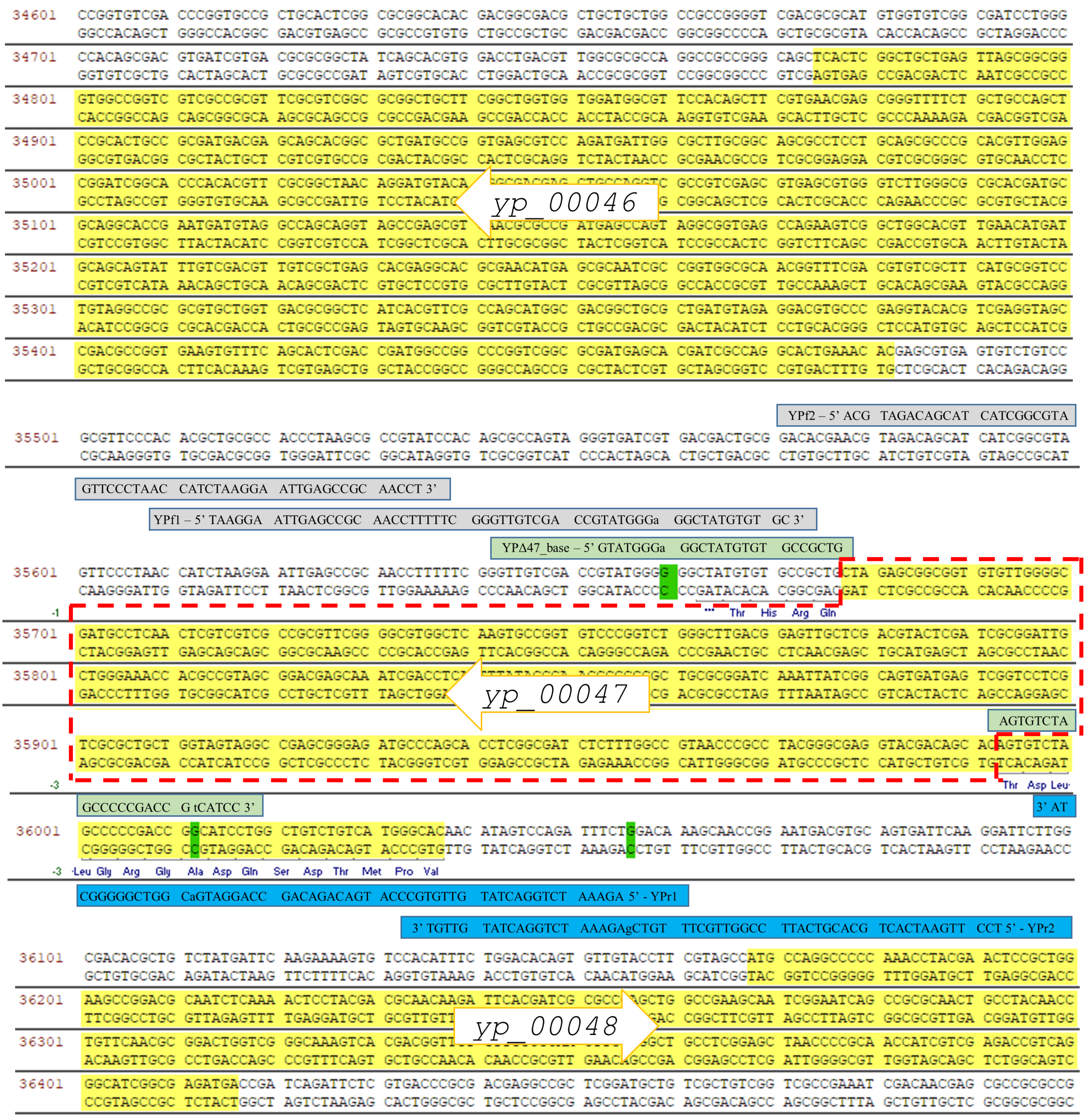

Figure S3. Schematic representation of the yp_00047 deletion construct.
Genes yp_00046, yp_00047 (deletion target), and yp_00048 are highlighted in yellow. The synthesized 50 bp base sequence (YPΔ47_base, light green) contains two 25 bp flanking regions adjacent to the deletion site, indicated by a red dashed line. Extender primers YPf1/YPf2 and YPr1/YPr2 are shown in gray and blue, respectively. Primer-introduced SNPs are marked in dark green.
